# Identification of the oncogenic role and clinical implication of *LZTS3* in Colon Adenocarcinoma

**DOI:** 10.7150/jca.102204

**Published:** 2025-01-01

**Authors:** Kui Ye, Huaxiang Cao, Zihao Xu, Peng Liu, Xueying Jiang, Qiancheng Liu, Hongquan Yang, Menghua Huo, Teng Wang, Jian Wang

**Affiliations:** 1 Department of Gastroenterology, Wuxi Second Geriatric Hospital, Wuxi Huishan Second People's Hospital, Wuxi, 214174, Jiangsu, China.; 2 Department of Gastrointestinal Surgery, Affiliated Hospital of Jiangnan University, Wuxi, 214122, Jiangsu, China.; 3 Department of Oncology, Geriatric Medical Center, Wuxi Second Geriatric Hospital, Wuxi Huishan Second People's Hospital, Wuxi, 214174, Jiangsu, China.; 4 Department of Oncology, Affiliated Hospital of Jiangnan University, Wuxi, 214122, Jiangsu, China.

**Keywords:** colorectal adenocarcinoma, *LZTS3*, prognosis, immune therapies

## Abstract

Colorectal carcinoma (CRC) is a highly prevalent and life-threatening disease with multi-stage progression, characterized by diverse molecular expression patterns at distinct stages, making treatment particularly challenging. Early detection and diagnosis of CRC are vital and can greatly benefit from the discovery of effective biomarkers. Researchers have identified novel gene signatures that play pivotal roles in specific CRC types or stages. Leucine Zipper Tumor Suppressor Family Member 3 (LZTS3), a well-known tumor suppressor in several cancer types such as lung carcinoma, has sparked interest. However, our comprehensive *in silico* studies revealed a contrasting role for LZTS3 in CRC. We found that LZTS3 was highly expressed in CRC cases and associated with poor clinical outcomes. Gene enrichment analyses indicated that LZTS3 expression levels correlate with immune checkpoint inhibition, emphasizing the therapeutic potential of LZTS3 for colorectal adenocarcinoma (COAD). In conclusion, our results suggest a potential oncogenic role of LZTS3 in colorectal cancers, challenging previous perceptions of its function. Further research is needed to explore the precise mechanisms underlying this unexpected role and its implications for CRC diagnosis and therapy.

## Background

Colorectal cancer (CRC) is a globally lethal cancer type. Notably, mainland China reported over 0.6 million new cases in 2019, marking it as the cancer with the highest incidence across all age groups 1. Despite early-stage screening and the adoption of novel therapies for CRC, there has been an upward trend in early-onset incidence in recent years. Unhealthy dietary habits and a lack of neoadjuvant therapies have been proposed as partial causes 2,3. As the predominant subtype of CRC, colon adenocarcinoma (COAD) poses significant treatment challenges, particularly in advanced stages. Drug resistance is becoming increasingly common, further complicating the management of COAD. Tumor cell invasion, migration, metastasis, and recurrence ultimately result in high mortality rates 4,5. Tumor suppressor genes encode proteins that control tumor growth, and the loss of tumor suppressors can cause abnormal cell proliferation. Frequent inactivation of tumor suppressor genes has been reported in patients with the microsatellite instability subtype of colon cancers 6,7. It's worth noting that under certain circumstances, tumor suppressors in one cancer type may function as oncogenes in another.

Leucine zipper tumor suppressor family member 3 (LZTS3) was previously proposed as a potential tumor suppressor in lung cancer and colon cancer 8,9. It has been shown that suppressing microRNA miR-1275 increases the expression level of LZTS3 and promotes cell growth ability and metastasis. However, mechanistic studies were not involved in these reports to demonstrate the functional role of LZTS3. The exact mechanism of how this gene affects tumor growth has not been well characterized. In a more recent expression microarray analysis, LZTS3 played a different role. Differential expression genes (DEGs) were first identified from 6 integrated CRC datasets, and LZTS3 was one of the upregulated DEGs, exhibiting adverse prognostic value in CRC 10. These inconsistent results reflect the complicated role and underlying mechanism of LZTS3 in CRC. In this present study, we used the most updated TCGA-COAD dataset to perform comprehensive analyses. Our aim is to decipher not only the clinical and functional role of LZTS3 but also its potential molecular mechanisms in COAD. We hope that this study will provide clinical implications and novel therapeutic insights into LZTS3 in COAD.

## Methods

### Samples and clinical information

The mRNA expression level and clinical information of COAD patients were acquired from The Cancer Genome Atlas Program (TCGA), including 483 COAD cases and 41 normal colorectal tissues.

### Pan-cancer analysis

We downloaded TCGA pan-cancer data from GEPIA database and compared the mRNA expression level of *LZTS3* between tumor samples and corresponding normal tissue samples. Statistical differences were defined as significant when the fold-changes (FC) >1 and *p* value below 0.01 The cutoff for evaluating statistical significance was set at 10%.

### Survival and prognostic analyses

TCGA-COAD samples (n = 455) were stratified into *LZTS3* high expression (*LZTS3*-high) and low expression (*LZTS3*-low) groups, using the optimal cut-off point. Kaplan-Meier plots were used to illustrate the overall survival (OS) and progression-free survival (PFS) status of these two groups. To evaluate the prognostic power of *LZTS3* in COAD, univariate analysis was performed between the indicated parameters and OS using the “survival” R package. Variables showing a significant association with OS were subjected to further investigation through a multivariate analysis, also conducted using the “survival” R package.

### Differential expression analysis

To profile the differential gene expressions (DEGs) between *LZTS3*-high samples and *LZTS3*-low colorectal samples, we employed the limma software to identify and cluster DEGs with a log2-fold-change (FC) > 2 and a false discovery rate (FDR) of less than 0.05.

### Functional enrichment analysis

Based on the median expression level of *LZTS3*, DEGs that fulfilled the requirement that an adjusted *p* value of lower than 0.05 and a log2-FC =1 were identified in two groups of TCGA-COAD samples (*LZTS3*-high and *LZTS3*-low) by using the limma package. Overlapping degree of the top ten DEGs and *LZTS3* was identified and corrected by Spearman's correlation analysis, and functional enrichments of DEGs were indicated by the clusterProfiler package. While Gene Ontology (GO) analysis identified the significant biological processes, Kyoto Encyclopedia of Genes and Genomes (KEGG) analysis indicated the prominent signaling pathways. On the other hand, Gene set enrichment analysis (GSEA) was performed in conjunction with the above analyses to see how the genes distributed in LZTS-3 high or low groups. Pathways with a high enrichment score, an adjusted *p* value of less than 0.05 was defined as significant.

### Prognostic prediction

Nomogram prediction model was used to estimate the time-dependent OS of COAD. The prediction power of nomogram was calibrated accordingly and the time-dependent sensitivities and specificities of the nomogram for OS were analyzed by the area under the curve (AUC) of ROC curve. Analysis was performed in the R environment. The rms package generated the nomogram and calibration plots, the timeROC package output the time-dependent ROC curve, and the Hmisc package of the R program. *P* < 0.05 indicates significant differences.

### Immune cell analysis

Tumor-Infiltrating Immune Cells (TIICs) in COAD samples were analyzed by CIBERSORT 11. Gene signature matrix of 22 TIICs were generated from the CIBERSORT platform.

### Drug sensitivity tests

IC_50_ represented the half inhibition efficiencies of indicated drugs was calculated by the “pRRophetic” R package. IC_50_ was compared between LZTS3-high and LZTS3-low groups, respectively.

### Immunohistochemistry (IHC) staining

The tissues underwent deparaffinization using xylene and rehydration through an alcohol gradient. The activity of endogenous peroxidase was inhibited by 3% H_2_O_2_ for a duration of 15 minutes. Antigen retrieval was performed by immersing the slides in a 10 mM sodium citrate buffer (pH 6.0) and maintaining them at a subboiling temperature for 10 minutes. The slice was then blocked with 10% goat serum for 1 hour, followed by overnight incubation at 4 °C in a humidified chamber with LZTS3 antibody (Affinity Biosciences). Subsequently, the slice was incubated with a secondary antibody labeled with horseradish peroxidase. The tissues were stained by diaminobenzideine and evaluated under a light microscope.

### Statistical analysis

R (v4.1.3, R Foundation for Statistical Computing, Vienna, Austria) was applied to examine statistical significance where appropriate. FDR was used to make adjustments for the multiple testing. Two-sided *p* values less than 0.05 was deemed as statistically significant.

## Results

### Profiling the mRNA expression levels of *LZTS3* in tumor and normal tissues

**Figure 1:**
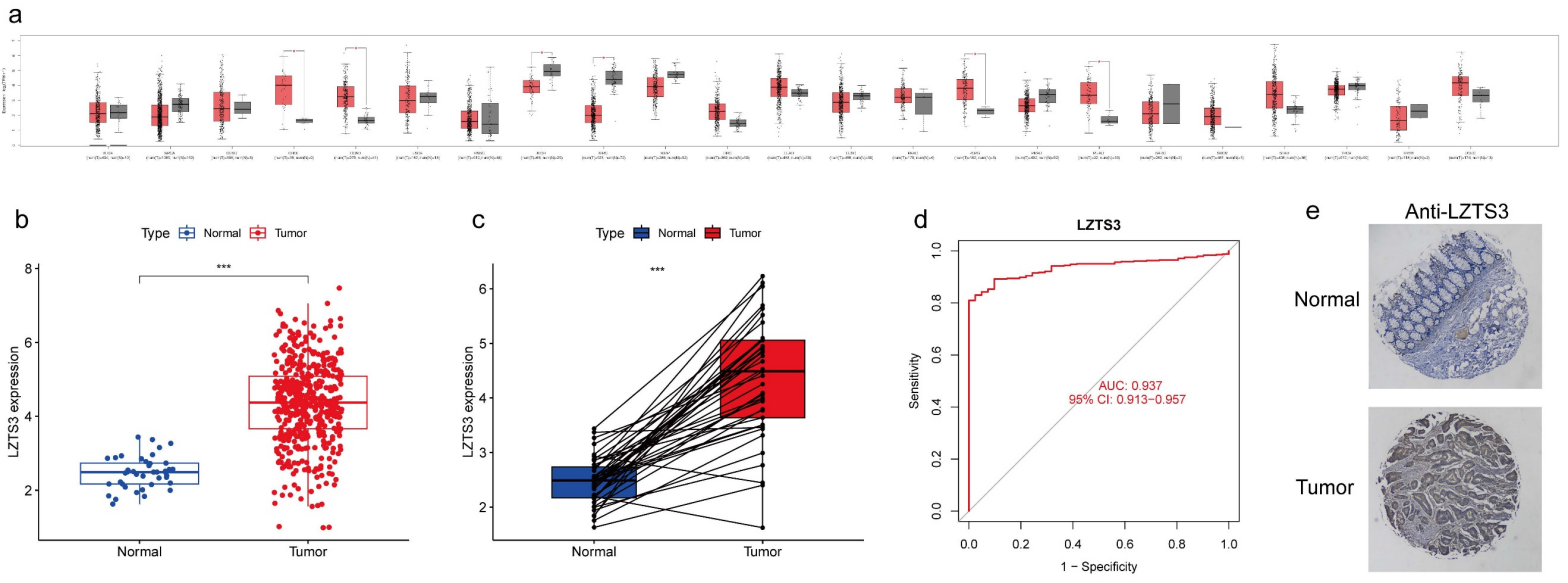
** Upregulation of LZTS3 in COAD.** a) Expression alterations of LZTS3 in different tumors. b) LZTS3 was significantly increased in COAD (n = 483, TCGA) compared with normal tissues (n = 41, TCGA). c) LZTS3 was increased in COAD samples compared with their adjacent normal tissues (n = 41, TCGA). d) LZTS3 expression is a potential diagnostic factor for COAD (TCGA cohort). ***, *p* < 0.001. e) Expression pattern of LZTS3 was examined by IHC staining in tissue microarray samples.

Despite the reported role of *LZTS3* as a tumor suppressor in a few cancer types, its expression pattern has not been fully investigated. To better understand the expression profiles of *LZTS3* in cancers, we analyzed its mRNA expression levels in the most common tumors and also compared with the levels in corresponding normal tissues, respectively. Among 23 tissue types, the mean value of *LZTS3* expression was decreased in eight of tumor types and increased in eight tumor types (Figure 1a). The different expression pattern of *LZTS3* implies diverse roles in tumors. In TCGA-COAD cases (n = 483), *LZTS3* expression was significantly higher than in unpaired colorectal normal (n = 41, Figure 1b) or paired adjacent normal tissues (Figure 1c). To further explore the diagnostic value of *LZTS3* in COAD, a ROC curve was applied to represent the diagnostic or prognostic value of this gene (Figure 1d). With an area under the curve (AUC) of 0.937 (95% CI: 0.913-0.957), *LZTS3* was a potential specific and sensitive biomarker for identifying COAD cases. In addition, in order to evaluate the expression of LZTS3 in terms of the protein level, we assessed LZTS3 expression by immunohistochemistry using a tissue array. It is obvious that the LZTS3 was significantly higher in tumor specimens than in normal specimens (Figures 1e).

### *LZTS3* expression correlates with advanced stages of COAD

**Figure 2:**
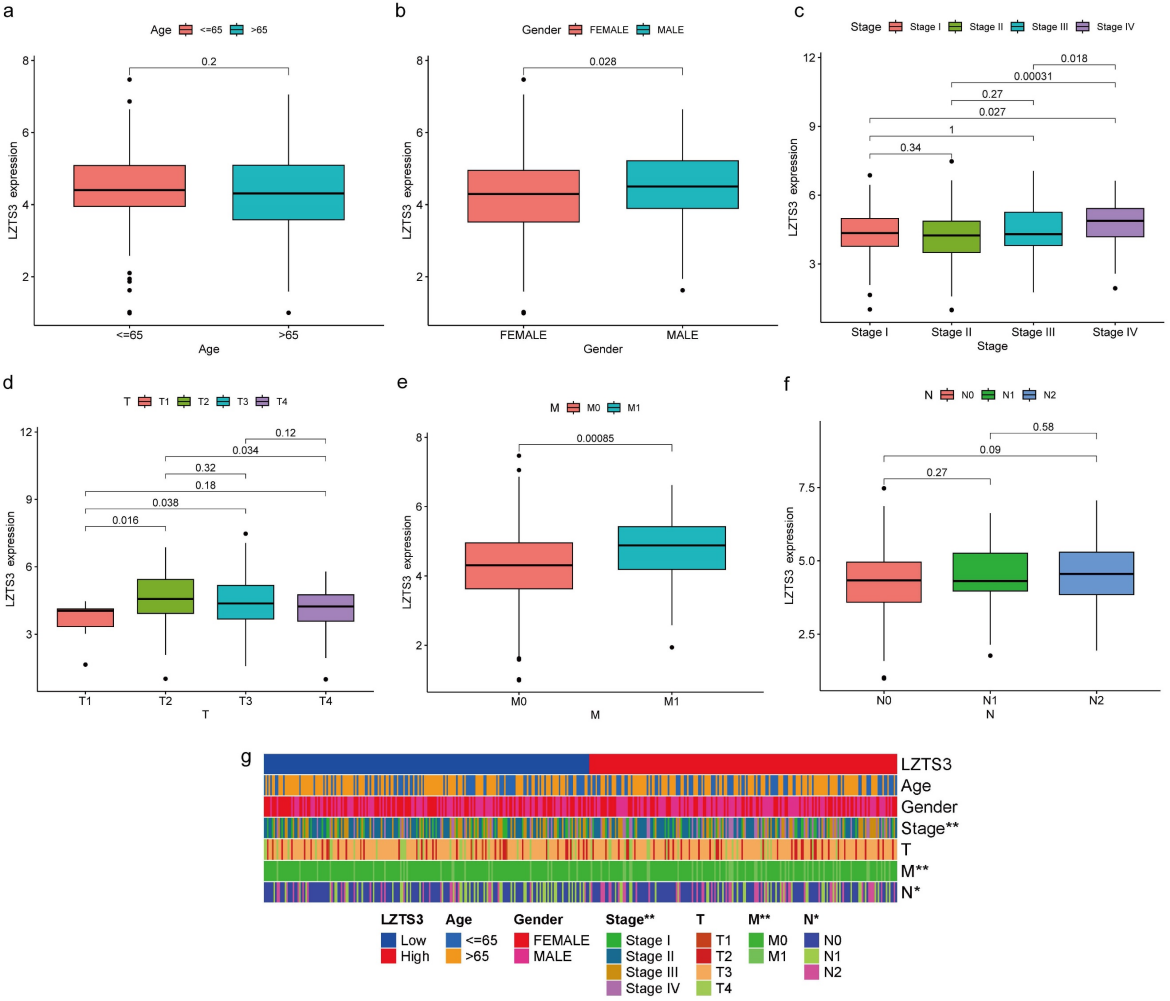
** Correlation between *LZTS3* expression and clinical features in COAD.** The clinical features include a) age, (b) gender, (c) advanced clinical stages, and (d-f) TMN stages. (g) Heatmap shows the patterns of indicated clinical features in COAD patients (TCGA cohort) with low or high expression of LZTS3. *, *p* < 0.05; **, *p* < 0.01.

As *LZTS3* expression was higher in COAD than normal tissues, we further explore whether high expression of this gene has potential clinical implications. We then delve into the correlation between *LZTS3* and several clinicopathological parameters. Firstly, *LZTS3* expression has no significant differences between patients older than 65 years and the younger counterparts (Figure 2a), but males have a significant higher level of *LZTS3* than females (Figure 2b). Next, we determine if *LZTS3* was associated with disease progression. In the first three stages of COAD, there are no significant changes of *LZTS3* expression. However, the *LZTS3* expression in stage IV was significant upregulated compared with the earlier three stages, respectively (Figure 2c). To assess whether *LZTS3* expression can serve as a predictor of metastasis, we scrutinized its expression pattern within the framework of the tumor-node-metastasis (TNM) staging system. As shown, *LZTS3* exhibited higher expression in the later phases of each classification, with a particular increase observed in the metastasis (M) stage (Figure 2d-f). This observation implies a strong association between *LZTS3* and advanced stages of the disease, suggesting its involvement in disease progression. Furthermore, we conducted a multiple correlation analysis to assess the relationship between *LZTS3* and the clinical characteristics. The heatmap clearly illustrates that *LZTS3* expression is significantly correlated with advanced stages, N stage, and M stage (Figure 2g). These findings suggest a potential clinical role for *LZTS3* in predicting tumor progression.

### *LZTS3* independently predicts worse clinical outcomes of COAD patients

**Figure 3:**
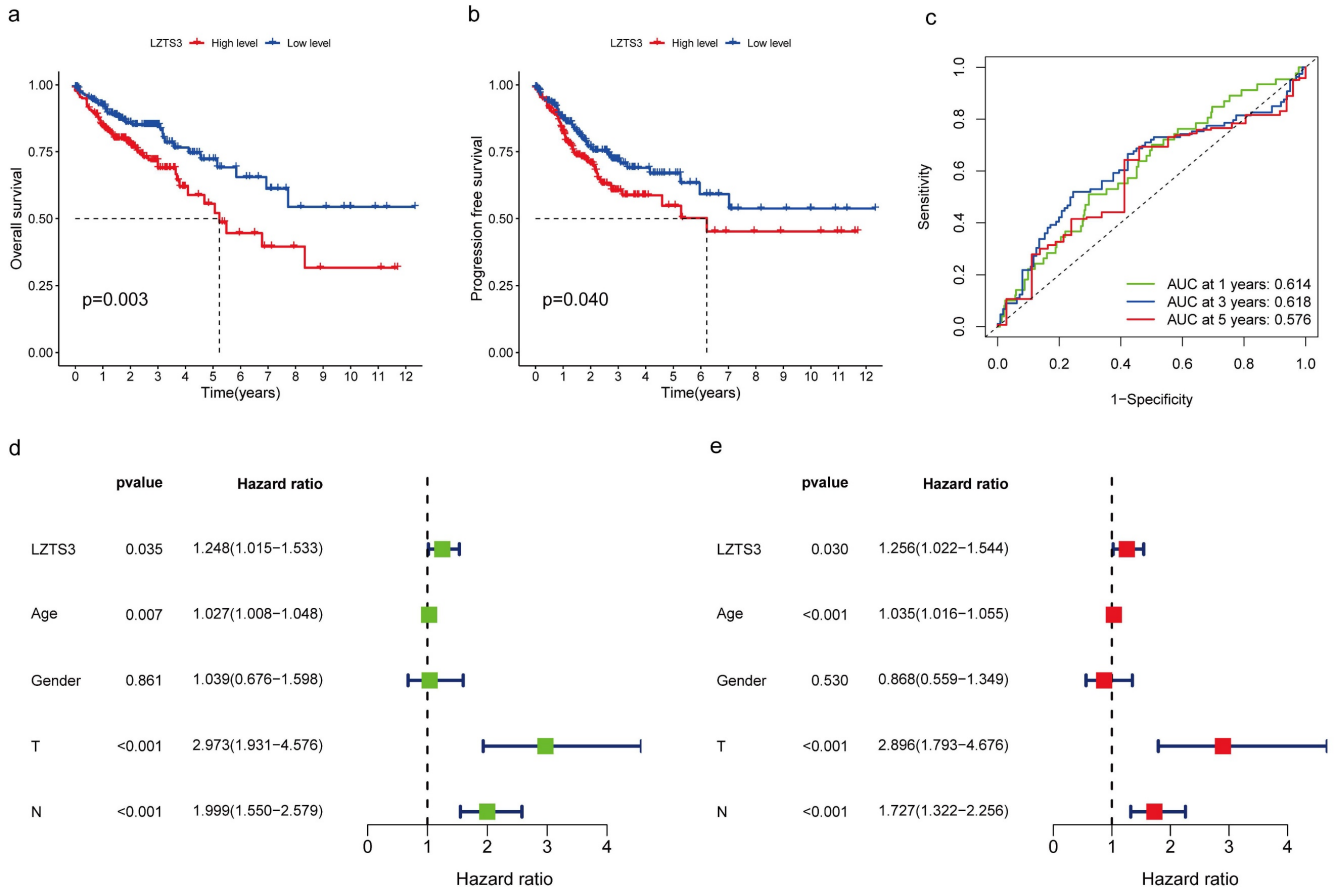
** Correlation between *LZTS3* expression and clinical outcomes in COAD.** The Kaplan-Meier survival curves represent the a) overall survival and b) progression-free survival in patients with high expression or low expression of *LZTS3* (TCGA cohort). c) The Area under the ROC curve (AUC) reflects the predictive power of *LZTS3* as a marker for the first, third, and fifth year of survival. d) Univariate and e) multivariate Cox regression analysis indicates that LZTS3 is independently correlated with OS of COAD patients (*p* < 0.05; TCGA cohort).

The correlation between *LZTS3* expression and survival status in COAD patients was analyzed by using the Kaplan-Meier survival curves. The analysis showed that high *LZTS3* mRNA expression level was significantly associated with worse overall survival (OS) (Figure 3a, *p* = 0.003) and worse progression-free survival (Figure 3b, *p* = 0.04) in COAD. As the area under the curve (AUC) for one, three and five years were all above 0.5, *LZTS3* represents a robust prognostic marker for predicting the clinical outcomes of COAD patients (Figure 3c). Additionally, the univariate forest plot reveals that *LZTS3* expression is one of the significant factors correlated to OS (*p* < 0.035; Hazard Ratio, HR, 1.248 (1.015-1.533)) (Figure 3d). According to the multivariate Cox regression analysis, *LZTS3* was shown as an independent prognostic marker (*p* < 0.035; HR, 1.256 (1.022-1.544)) (Figure 3e).

**Figure 4:**
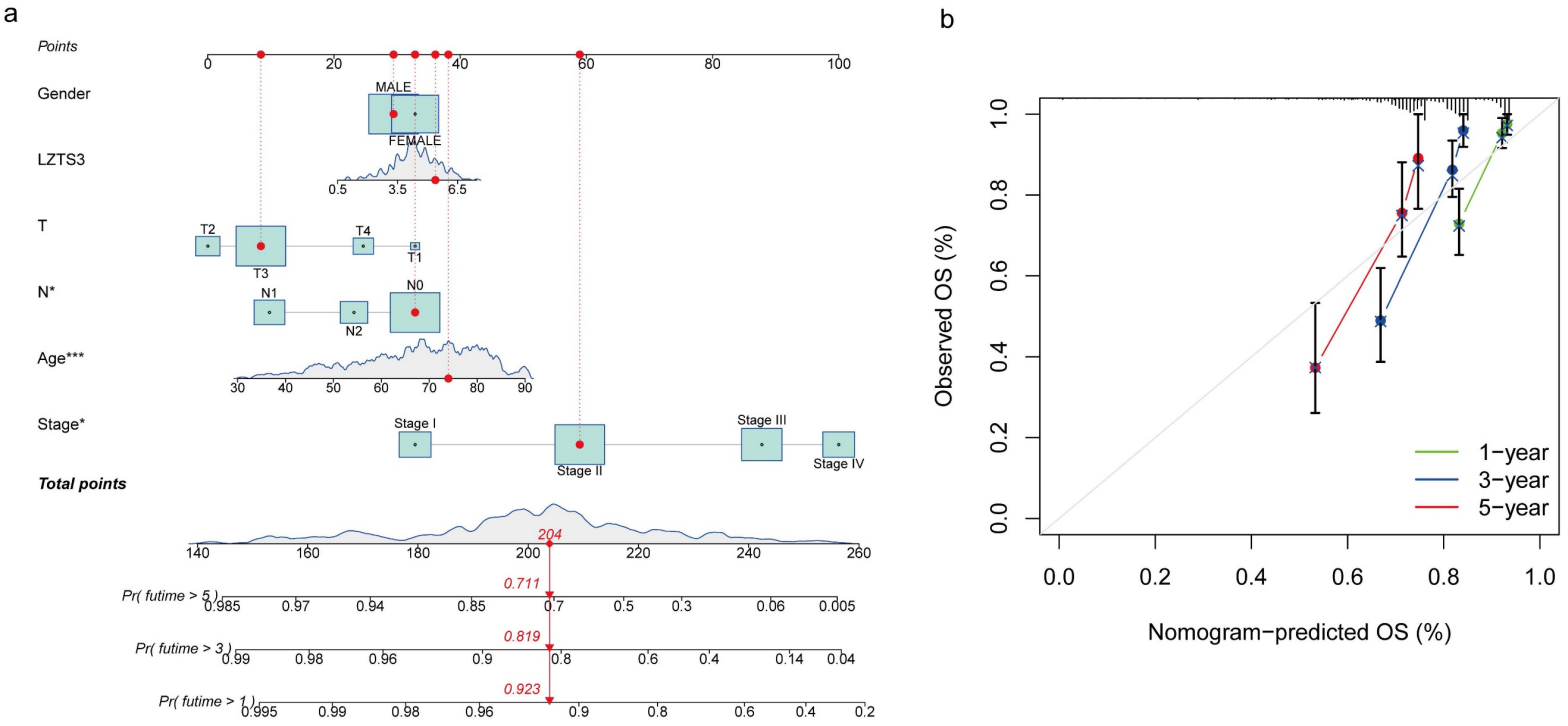
** The prognostic potential of LZTS3 in COAD overtime is determined by the nomogram.** a) Nomogram model predicts the first-, third-, and fifth-year OS in COAD patients with high expression of *LZTS3*. *, *p* < 0.05, ***, *p* < 0.001. b) Calibration curve for the OS nomogram model in COAD patients (TCGA cohort). A dashed diagonal line indicates the ideal nomogram.

In addition to utilizing the TNM staging system, we employed a prognostic nomogram to predict the OS probability in advanced COAD patients from the TCGA cohort. Points were assigned for each identified risk factor by drawing lines upward from the corresponding values to the line of points on top. The sum of all these points provided the total points (upper panel, Figure 4a). As illustrated in this graph, a case with a total of 204 points corresponds to OS probabilities of 92.3% at one year, 81.9% at three years, and 71.1% at five years (lower panel, Figure 4a). We generated a calibration curve for one, three, and five years to evaluate the concordance between the predicted and actual overall survival (OS) probabilities. In the plots, the diagonal gray line signifies the actual OS probability, while the solid black line represents the OS probability predicted by the nomogram model. All three lines closely approximate the diagonal gray line, signifying that the nomogram model provides highly accurate estimations within this COAD cohort (Figure 4b). These combined analyses strongly emphasize the independent prognostic capability of *LZTS3* in COAD.

### *LZTS3* plays potential roles in tumor progression of COAD

**Figure 5:**
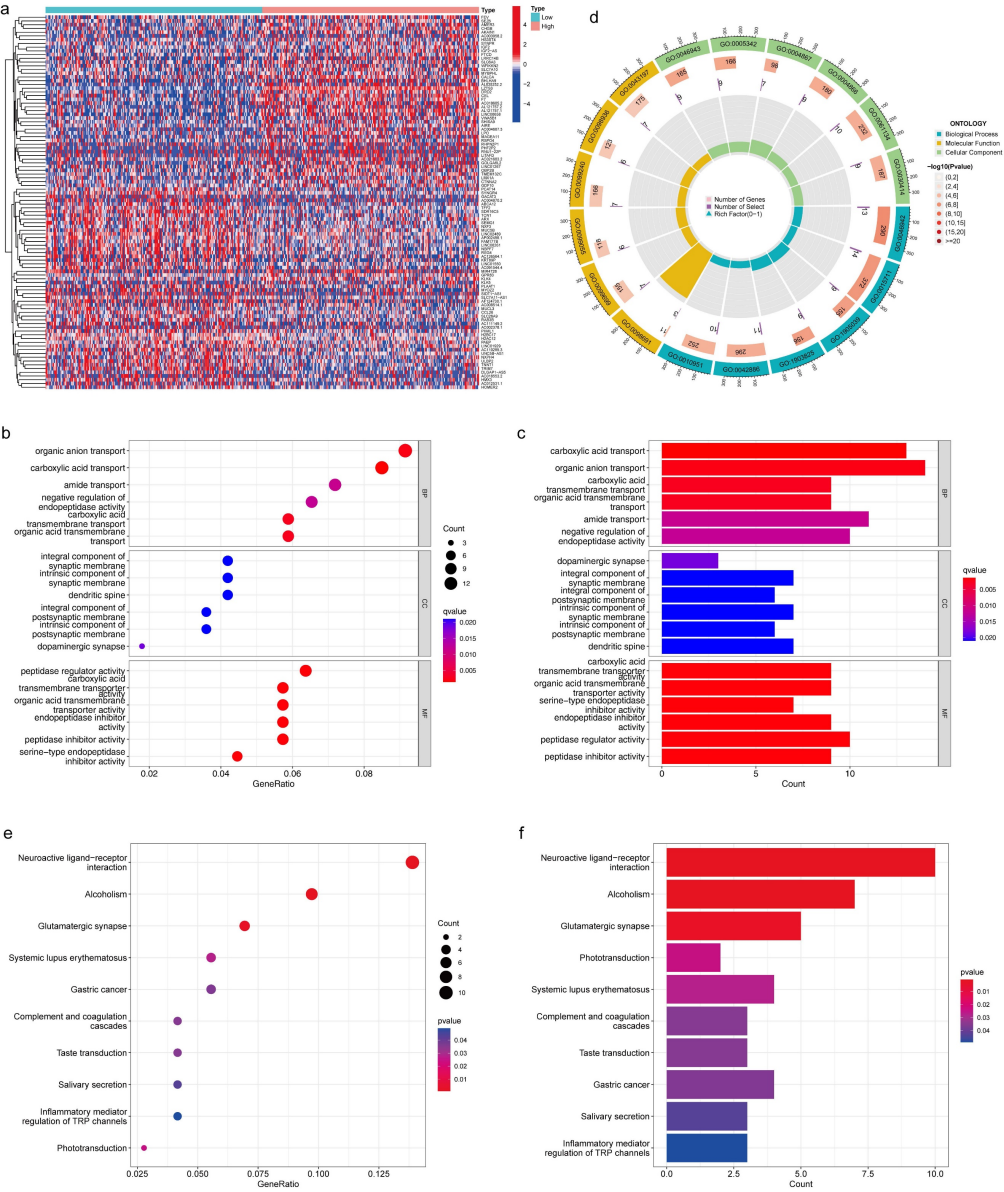
** GO and KEGG analyses reveal the potential role of *LZTS3* in COAD.** a) Heatmap shows the significant DEGs that relates to *LZTS3* expression. Graphs illustrate the significantly altered b-d) biological processes and e-f) KEGG signaling pathways in COAD patients (TCGA cohort) with high *LZTS3* expression.

We investigated the functional role of *LZTS3* in COAD patients by using the TCGA cohort. Based on the medium expression level of *LZTS3*, samples were divided into high expression and low expression groups, and DEGs with positive or negative correlation with *LZTS3* were clustered under each sample (*P* < 0.001, Figure 5a). Then we performed gene enrichment analyses to further reveal the potential functions of *LZTS3* in COAD. Gene ontology (GO) analysis indicated that the *LZTS3*-related DEGs were highly enriched in several cancer cell metabolic biological processes (BPs), including the organic anion transport, carboxylic acid transport, amide transport, negative regulation of endopeptidase activity, and organic acid transmembrane transport (*P* < 0.001, Figure 5b-d). On the other hand, these DEGs were particularly involved in the Neuroactive ligand-receptor interaction, Alcoholism, and Glutamatergic synapse signaling pathways (Figure 5e-f). To comprehensively characterize the functions of *LZTS3* in COAD, gene set enrichment analysis (GSEA) was performed.

**Figure 6:**
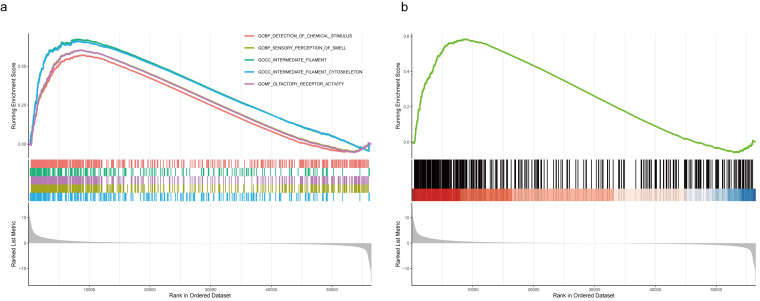
** GSEA indicates the potential function of *LZTS3* in COAD.** a) GO biological processes and b) KEGG signaling analyses indicates that LZTS3 upregulation is related to the olfactory receptor activity and transduction in COAD (TCGA cohort).

Several tumor-related hallmarks had significantly enrichment in the COAD samples with high expression of *LZTS3*, such as the GOMF_OLFACTORY_RECEPTOR_ACTIVITY (Figure 6a). The KEGG signaling analysis also indicated that the KEGG_OLFACTORY_TRANSDUCTION pathway was significantly associated with *LZTS3* in COAD (Figure 6b). Olfactory receptors (ORs) contribute to cell proliferation, migration, and secretion, olfactory transduction is recognized as an important biomarker for multiple types of carcinomas 12. Considering the expression level of *LZTS3* correlates with tumor progression, gene enrichment analyses suggest that *LZTS3* may have potential roles in regulating tumor progression.

### *LZTS3* relates to the tumor-infiltrating immune cell (TIIC)

**Figure 7:**
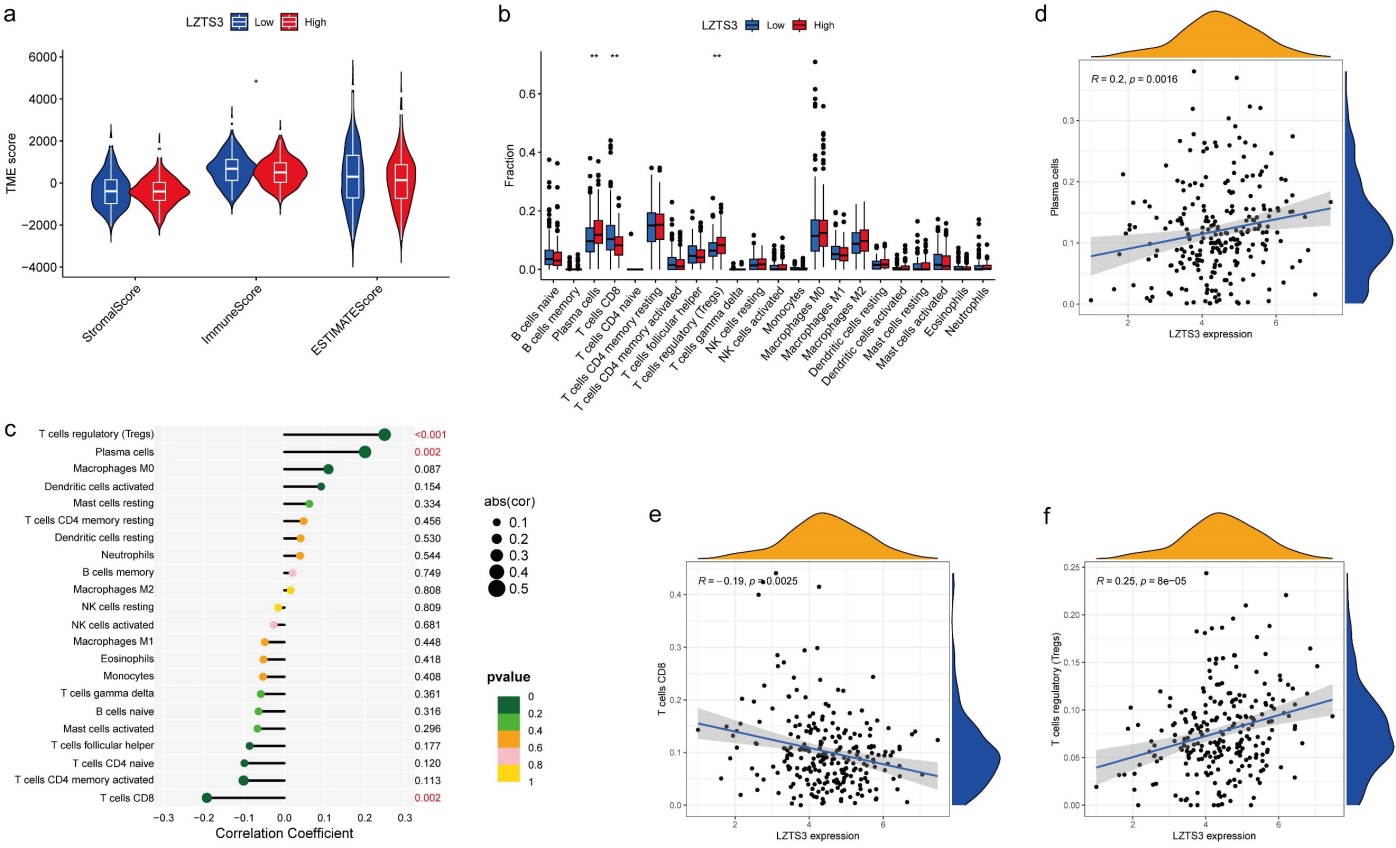
***LZTS3* expression is correlated with immune cell alterations in COAD.** a) *LZTS3* is significantly correlated with ImmuneScore rather the other two TME scores. b-c) For the major immune cell types, *LZTS3* is significantly correlated with plasma cells, Treg cells, and with CD8^+^ T cells. d-f) Correlation analysis of *LZTS3* expression and the proportion of three cell types in COAD. *, *p* < 0.05, **; *p* < 0.01; ***, *p* < 0.001.

The constituents of the tumor microenvironment (TME) wield substantial influence upon tumor progression and the efficacy of therapeutic interventions. To elucidate the potential role of *LZTS3* in TME, we conducted a comprehensive analysis, establishing correlations between three primary TME phenotypes and the expression levels of *LZTS3* in COAD samples sourced from TCGA. Our analysis indicated that high levels of *LZTS3* expression displayed a diminished Immune Score relative to their low *LZTS3* counterparts, however, no statistically significant differences were observed for the Stromal Score and the ESTIMATE Score (Figure 7a), thus implying a potential association between *LZTS3* expression and immune cells in COAD. Notably, the profile of TIICs impacts the clinical outcomes and therapeutic efficacy in COAD. To validate whether *LZTS3* is involved in immune cell redistribution in COAD, we conducted a comparative analysis (CIBERSORT) of the fractions of general immune cell populations in patients with high or low levels of *LZTS3*. Our findings demonstrate that *LZTS3*-high samples display a reduction in CD8^+^ T cells and an elevation in plasma cells and regulatory T (Treg) cells (Figure 7b). Importantly, the correlation between *LZTS3* expression and these three altered immune populations is statistically significant (Figure 7c), positive for plasma cells and regulatory T cells, but inversely related to CD8^+^ T cells (Figure 7d-f).

### The therapeutic potentials of *LZTS3* in COAD

**Figure 8:**
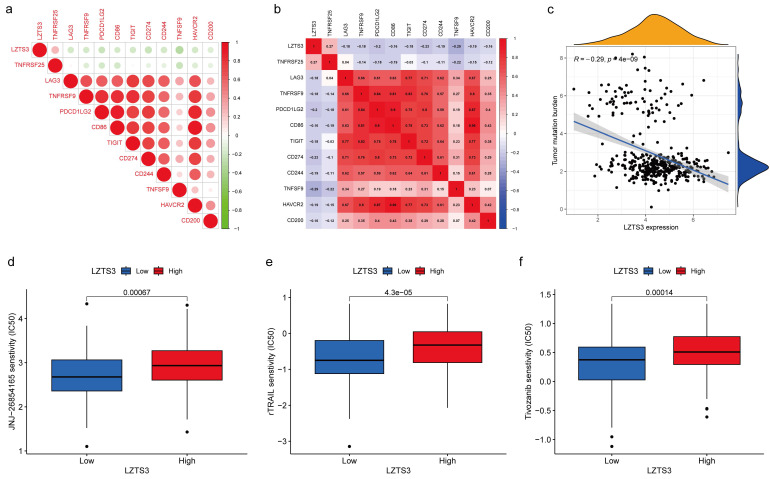
** The therapeutic potential of LZTS3 in COAD.** a, b) The association between *LZTS3* expression and immune checkpoint genes. c) *LZTS3* expression was negatively associated with tumor mutation burden. d) IC_50_ values for three targeted drugs were increased in COAD patients with high expression of *LZTS3*.

In TME, immune checkpoints play crucial roles in regulating tumor progression and understanding the underlying molecular mechanisms render therapeutic opportunities to COAD patients. For instance, immune checkpoint genes (ICGs) have been identified as novel drug targets for precision medicine. Regarding the connection between *LZTS3* and tumor immune cells, we next analyzed the expressional correlation between *LZTS3* and ICGs in COAD samples. Of the 11 selected ICGs, *LZTS3* exhibited a notable positive correlation with TNFRSF25 (Figure 8a-b). It's worth noting that TNFRSF25 has been identified as an unfavorable risk factor in CRC 13, although its precise role remains to be elucidated. On the other hand, tumor mutation burden (TMB) is characterized by somatic gene variations identified per million bases of genomic DNA. Elevated TMB serves as an indicator signifying favorable responses to immune checkpoint blockade therapies 14. As in Figure 8c, *LZTS3* exhibits an inverse relationship with TMB, underscoring the potential of targeting *LZTS3* as a promising strategy for successful immunotherapy.

As to the combinational therapies, we compared the sensitivity of three commonly used drugs in targeted therapies between *LZTS3*-high and *LZTS3*-low samples achieving from the Genomics of Drug Sensitivity in Cancer (GDSC) database. Drug sensitivity is represented by the value of half maximal inhibitory concentration (IC_50_). rTRAIL, JNJ-26854165, and Tivozanib all showed significantly higher value of IC_50_ in the *LZTS3*-high group than the *LZTS3*-low group (Figure 8d-f), implying that high expression of *LZTS3* is related to the treatment outcome of these three drugs.

## Discussion

This bioinformatic study unveils diverse expression patterns of *LZTS3* across various tissues, with its expression changes in different tumor types showing inconsistency. While some tumors exhibit reduced *LZTS3* levels, others demonstrate increased or no significant changes. This raises the question of whether LZTS3 plays distinct functional roles in different tumors. A previous report suggested that LZTS3 shares similarities with the tumor suppressors LZTS1 and LZTS2, leading to the suspicion that LZTS3 may have concurrent tumor-suppressive effects in CRC 9. Their validation was solely performed using quantitative RT-PCR, and it was limited in sample scale. When we analyzed *LZTS3* mRNA expression levels in the TCGA-COAD cohort, we obtained different results. *LZTS3* was found to be overexpressed in COAD samples in comparison to both paired and unpaired normal counterparts. IHC staining confirmed that the protein level of LZTS3 was significantly higher in COAD samples than in normal samples. Furthermore, upregulation of LZTS3 was notably associated with advanced tumor stages and poorer survival rates. These results suggest that LZTS3 independently poses a prognostic risk in COAD, although the ROC curve indicates that LZTS3 is not an outstanding prognostic marker. These findings align with the outcomes of a recent bioinformatic study 10. However, the specific transcriptional and post-transcriptional mechanisms responsible for LZTS3 upregulation in CRC remain to be elucidated. To the best of our knowledge, *LZTS3* mutations are not linked to cancer development. Missense mutations of *LZTS3* are present in a fraction of TCGA-COAD cases, but whether these mutations are associated with COAD development requires further investigation.

In terms of clinical implications, *LZTS3* appears to be a potential oncogenic factor in COAD. We identified a set of DEGs by comparing *LZTS3*-high and -low samples. These genes are primarily enriched in biological processes and signaling pathways that play crucial roles during tumor progression. These findings provide significant evidence to support the idea that *LZTS3* may indeed have oncogenic functions in the progression of COAD. To confirm the oncogenic role of *LZTS3*, further validation using various biological models is necessary. If the knockdown of *LZTS3* in COAD cell lines with relatively higher levels of *LZTS3* can inhibit their proliferation, migration, and invasion abilities, then the restoration of *LZTS3* in these cells might potentially rescue these oncogenic properties.

Our study reveals a correlation between *LZTS3* and tumor immune cells. Tumor mutation burden and tumor-infiltrating lymphocytes are two biomarkers known to influence immune checkpoint inhibition (ICI) 15. *LZTS3* expression is related to the reduction of the cytotoxic CD8^+^ T cells, the early effector playing anti-cancer effects 16,17, and increase of Treg cells, an CD4^+^ T subpopulation that contributes to tumor immune escape 18,19. This finding can correspond to the above demonstration that *LZTS3* expression is associated with and tumor progressions. Moreover, CD8^+^ T cell infiltration is dispensable for ICI 20-22, rescuing CD8^+^ T cells is a suggestive therapeutic option for the success of ICI treatment. Further investigations are needed to determine whether *LZTS3* could be a biomarker to evaluate the outcome after ICI treatment, and if targeting *LZTS3* could improve the ICI efficacy in CRC.

The first-line treatment for metastatic CRC typically combines chemotherapy and targeted therapy. This treatment regimen includes fluoropyrimidines as a crucial component, along with a selection of targeted small molecules or antibodies 23. In our study, we conducted correlation analyses with three representative targeted drugs. We found that elevated *LZTS3* expression is associated with reduced drug sensitivity in patients, indicating a potential risk when considering specific combination therapies. However, to further confirm the role of LZTS3 in CRC management, a larger sample cohort and a more extensive panel of drugs are needed for comprehensive validation.

## Conclusions

The current study suggests that *LZTS3* expression in COAD patient tissues is significantly elevated compared to normal tissues. This upregulation is associated with advanced disease stages and holds prognostic value. *LZTS3* expression is also linked to immune cell distributions, providing new therapeutic insights into immune checkpoint inhibition and first-line chemo-targeted combinational therapies.
